# Supplementary material for: Antimicrobial Resistance and Phylogenetic Relatedness of Salmonella Serovars in Indigenous Poultry and Their Drinking Water Sources in North Central Nigeria
Source: Microorganisms. 2024 Jul 26;12(8):1529. doi: 10.3390/microorganisms12081529 (PMC11356752; doi:10.3390/microorganisms12081529)
Supplement: Supplementary file 1 [file microorganisms-12-01529-s001.zip › microorganisms-3112220-supplementary.pdf]

Table S1a: Occurrence of *Salmonella* spp. in faeces of indigenous poultry from 15 markets in 3 states in North Central Nigeria

State	Market	No. of faecal samples tested	No. of faecal samples positive	Prevalence of *Salmonella*	95% Confidence interval
Benue	Otukpo	68	6	8.8%	0.04-0.19
	Modern	70	6	8.6%	0.04-0.19
	Wurukum	69	4	5.8%	0.02-0.15
	Gboko	67	5	7.5%	0.03-0.17
	Yandev	68	3	4.4%	0.01-0.13
	Total Benue markets	342	24	7.0%	0.05-0.10
Kwara	Sango	77	5	6.5%	0.02-0.15
	Oja-Oba	79	4	5.1%	0.02-0.13
	Ipata	76	4	5.3%	0.02-0.14
	Share	78	5	6.4%	0.02-0.15
	Oke-Oyi	78	6	7.7%	0.03-0.17
	Total Kwara markets	388	24	6.2%	0.04-0.09
Plateau	Mangu	75	7	9.3%	0.04-0.19
	Bokkos	76	3	3.9%	0.01-0.12
	Shendam	76	8	10.5%	0.05-0.20
	Yankaji	75	4	5.3%	0.02-0.14
	Kugiya	76	5	6.6%	0.02-0.15
	Total Plateau markets	378	27	7.1%	0.05-0.10
**Total**	**All markets**	**1,108**	**75**	**6.8%**	**5.4 to 8.4%**

Table S1b: Occurrence of *Salmonella* spp. in indigenous poultry drinking water sources in 15 markets in 3 states in North Central Nigeria

State	Market	No. of water samples tested	No. of water samples positive	Prevalence of *Salmonella*	95% Confidence interval
Benue	Otukpo	12	1	8.3%	0.00-0.40
	Modern	10	1	10%	0.00-0.46
	Wurukum	11	0	0%	0.00-0.32
	Gboko	13	0	0%	0.00-0.28
	Yandev	11	0	0%	0.00-0.33
	Total Benue markets	57	2	3.5%	0.00-0.13
Kwara	Sango	3	0	0%	0.00-0.69
	Oja-Oba	3	0	0%	0.00-0.69
	Ipata	5	0	0%	0.00-0.54
	Share	4	0	0%	0.00-0.60
	Oke-Oyi	5	1	20%	0.01-0.70
	Total Kwara markets	20	1	5.0%	0.00-0.30
Plateau	Mangu	7	0	0%	0.00-0.44
	Bokkos	4	0	0%	0.00-0.60
	Shendam	5	0	0%	0.00-0.54
	Yankaji	5	0	0%	0.00-0.53
	Kugiya	4	0	0%	0.00-0.60
	Total Plateau markets	23	0	0%	0.00-0.18
**Total**	**All markets**	**100**	**3**	**3.0%**	0.65 to 8.8%

Table S2: Table showing the serovars of *Salmonella* spp. strains isolated from sampled indigenous poultry droppings and poultry drinking water sources in North Central Nigeria

S/No	*Salmonella* serovars	Isolates from poultry droppings	Isolates from water	Total isolates	Serovar (%)
1	Chester	4	2	6	14
2	Agama	4	0	4	9
3	Isangi	4	0	4	9
4	Offa	3	0	3	7
5	Derby	2	1	3	7
6	Saintpaul	2	0	2	5
7	Laredo	2	0	2	5
8	Give	2	0	2	5
9	Orion	2	0	2	5
10	Monophasic Variant of *Salmonella* Typhimurium	2	0	2	5
11	Widemarsh	2	0	2	5
12	Typhimurium	1	0	1	2
13	Telelkebir	1	0	1	2
14	Durham	1	0	1	2
15	Larochelle	1	0	1	2
16	Kingston	1	0	1	2
17	Vom	1	0	1	2
18	Linguere	1	0	1	2
19	Bareilly	1	0	1	2
20	Lansing	1	0	1	2
21	Luedinghausen	1	0	1	2
22	*Salmonella* enterica subspp enterica 6,7:c-	1	0	1	2
23	*Salmonella* enterica subspp enterica 6,7: a-	1	0	1	2
**Total**	**23**	**41**	**3**	**44**	**100**

Table S3: ***Salmonella*** **strains for Chicken, Duck, Turkey and Water and their accession numbers**

Base name	Serovars	Sample source	Biosample Number	Accession Number
23-43780_S11	S. Give	Chicken droppings	SAMN41784839	JBEQJK000000000
23-43783_S12	S. Give	Chicken droppings	SAMN41784840	JBEQJJ000000000
23-43786_S13	S. Offa	Chicken droppings	SAMN41784841	JBEQJI000000000
23-43788_S14	S. Offa	Chicken droppings	SAMN41784842	JBEQJH000000000
23-43794_S15	S. Chester	Water	SAMN41784843	JBEQJG000000000
23-43796_S16	S. Chester	Chicken droppings	SAMN41784844	JBEQJF000000000
23-43804_S17	S. Widemarsh	Chicken droppings	SAMN41784845	JBEQJE000000000
23-43805_S18	S. Widemarsh	Chicken droppings	SAMN41784846	JBEQJD000000000
23-43807_S19	S. Agama	Chicken droppings	SAMN41784847	JBEQJC000000000
23-43808_S20	S. Agama	Turkey droppings	SAMN41784848	JBEQJB000000000
23-43809_S21	S. Agama	Chicken droppings	SAMN41784849	JBEQJA000000000
23-43810_S22	S. Agama	Chicken droppings	SAMN41784850	JBEQIZ000000000
23-43813_S23	S. Saintpaul	Chicken droppings	SAMN41784851	JBEQIY000000000
23-43815_S24	S. Saintpaul	Chicken droppings	SAMN41784852	JBEQIX000000000
23-43819_S25	S. Derby	Water	SAMN41784853	JBEQIW000000000
23-43821_S26	S. Derby	Chicken droppings	SAMN41784854	JBEQIV000000000
23-43822_S27	S. Derby	Chicken droppings	SAMN41784855	JBEQIU000000000
23-43825_S28	S. Orion	Chicken droppings	SAMN41784856	JBEQIT000000000
23-43827_S29	S. Orion	Chicken droppings	SAMN41784857	JBEQIS000000000
23-43828_S1	S. Isangi	Chicken droppings	SAMN41784858	JBEQIR000000000
23-43829_S2	S. Isangi	Chicken droppings	SAMN41784859	JBEQIQ000000000
23-43833_S3	S. Laredo	Chicken droppings	SAMN41784860	JBEQIP000000000
23-43834_S4	S. Laredo	Chicken droppings	SAMN41784861	JBEQIO000000000
23-43836_S5	S. Luedinghausen	Chicken	SAMN41784862	JBEQIN000000000

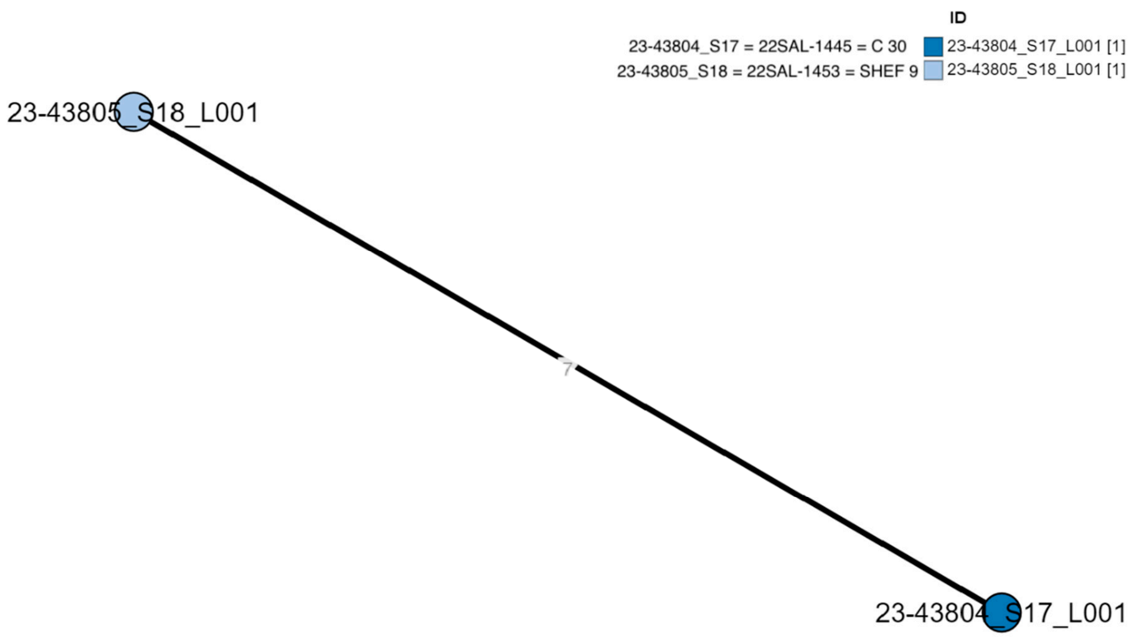

Figure S1: Cluster analysis for *Salmonella* Widemarsh isolated from poultry droppings from 2 markets in Plateau State

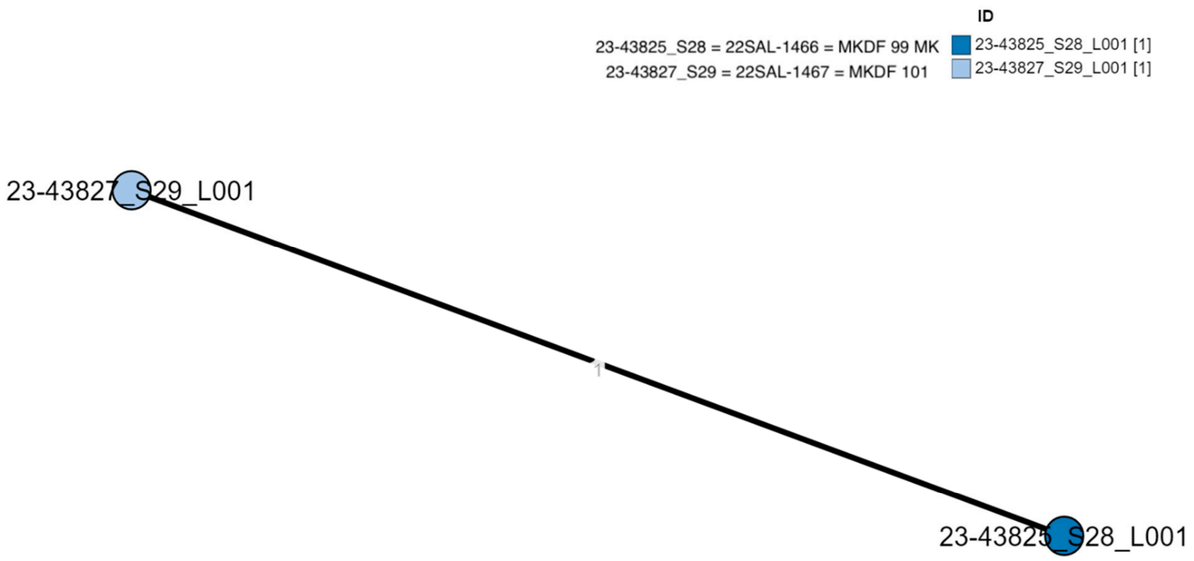

Figure S2: Cluster analysis for *Salmonella* Orion isolated from poultry droppings in 2 markets in Benue State

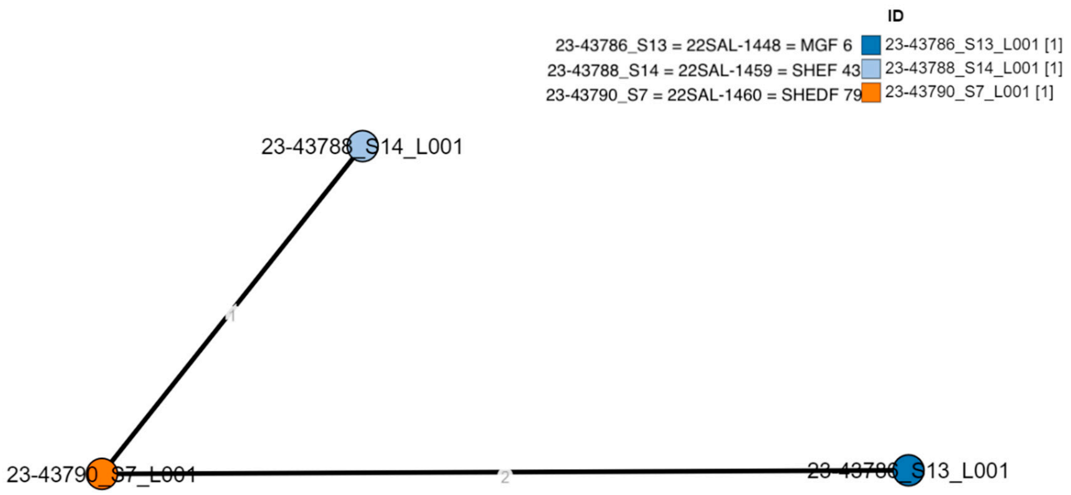

Figure S3: Cluster analysis for *Salmonella* Offa isolated from poultry droppings in 3 markets in Plateau State

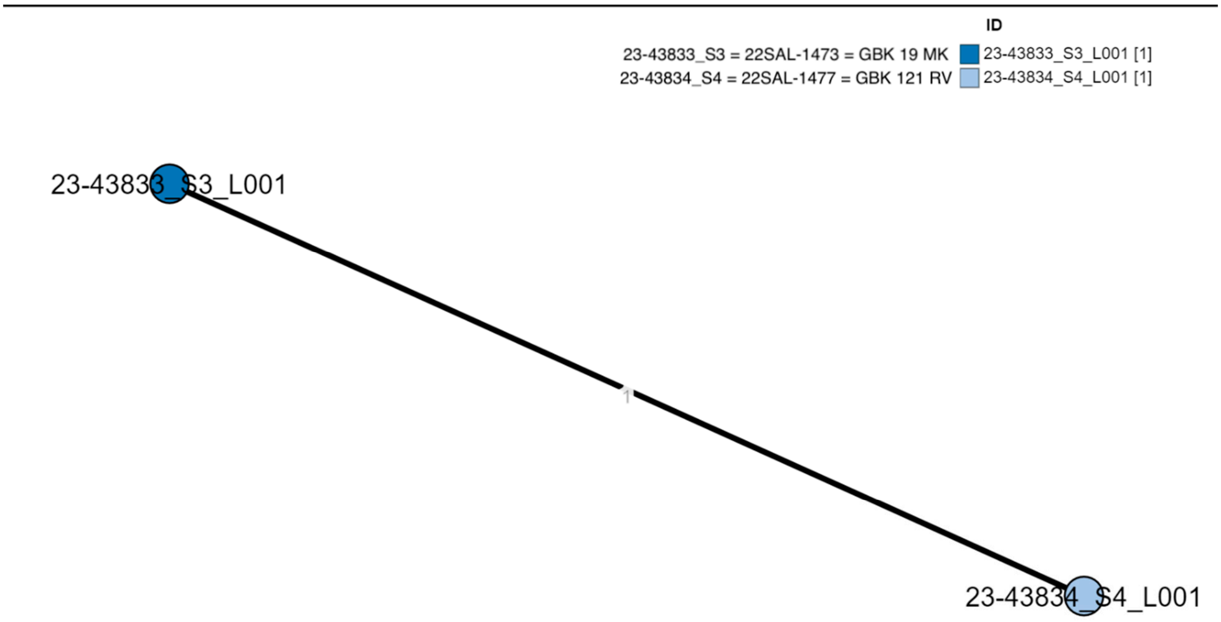

Figure S4: Cluster analysis for *Salmonella* Laredo isolated from poultry droppings in 2 markets in Benue State

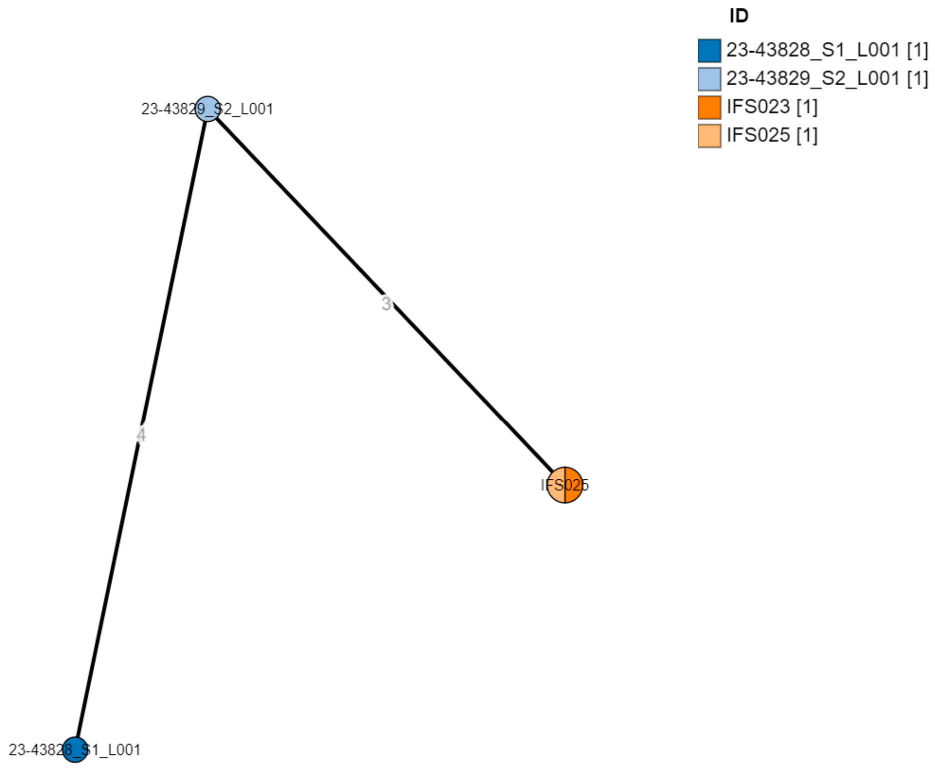

Figure S5: Cluster analysis for *Salmonella* Isangi isolated from poultry droppings in 3 markets (2 in Benue and 1 in Plateau States)

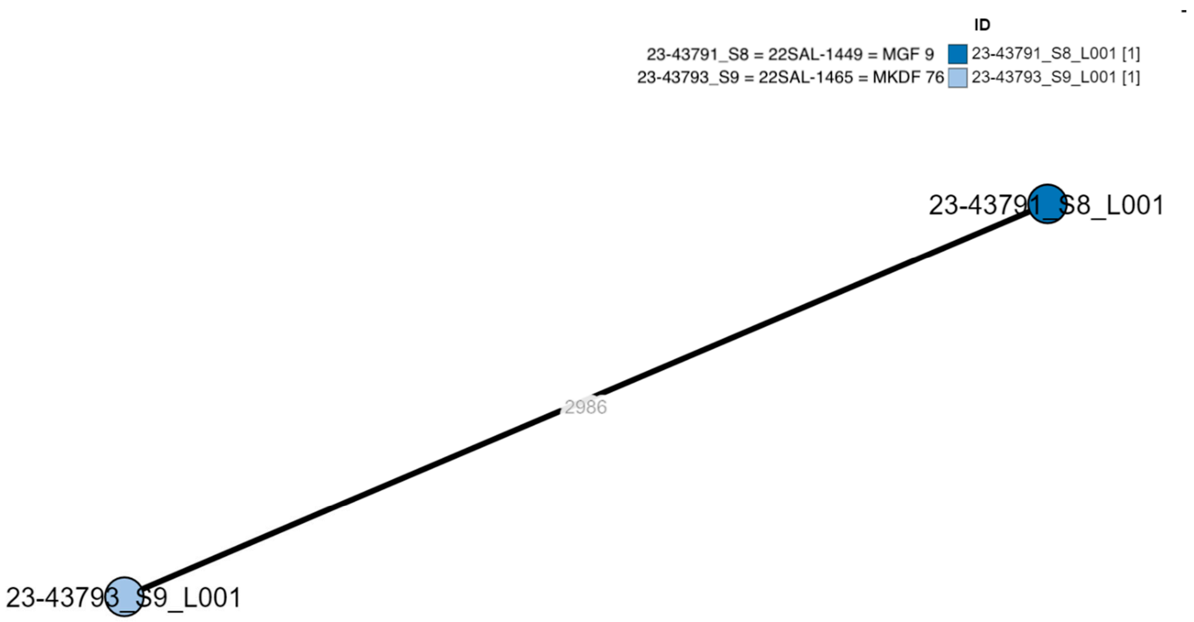

Figure S6: Cluster analysis for Monophasic Variant of *Salmonella* Typhimurium isolated from poultry droppings in 2 markets in Benue and Plateau States

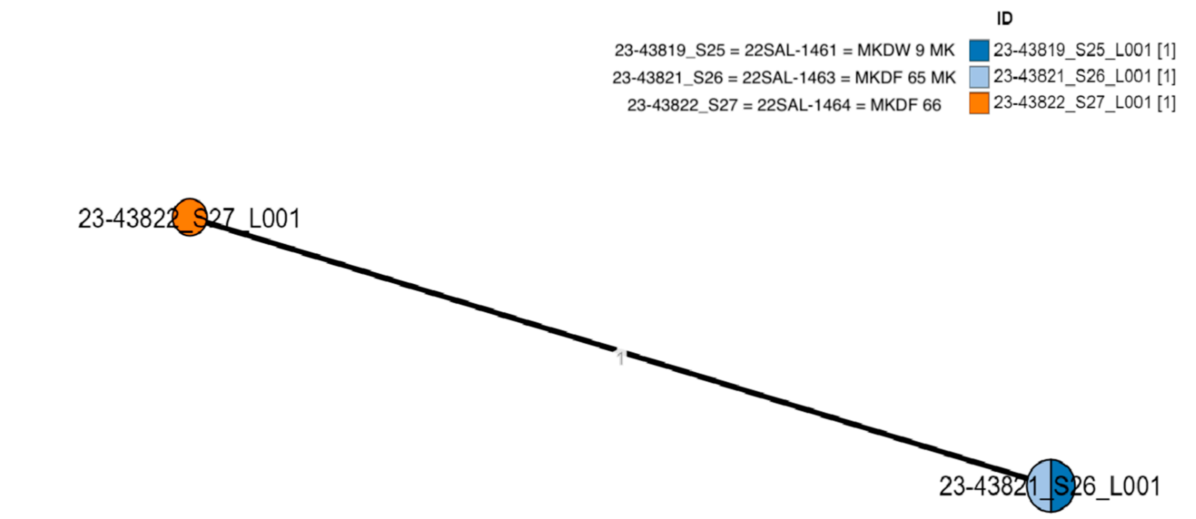

Figure S7: Cluster analysis for *Salmonella* Derby isolated from poultry droppings and poultry drinking water source in 1 market in Benue State

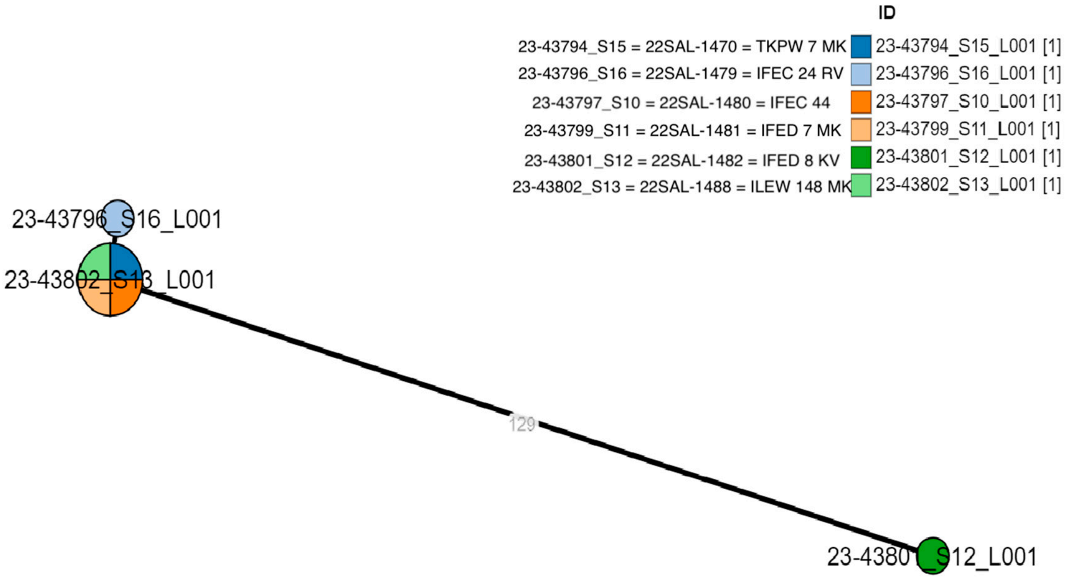

Figure S8: Cluster analysis for *Salmonella* Chester isolated from poultry droppings and poultry drinking water source in 3 markets in Benue and Kwara States

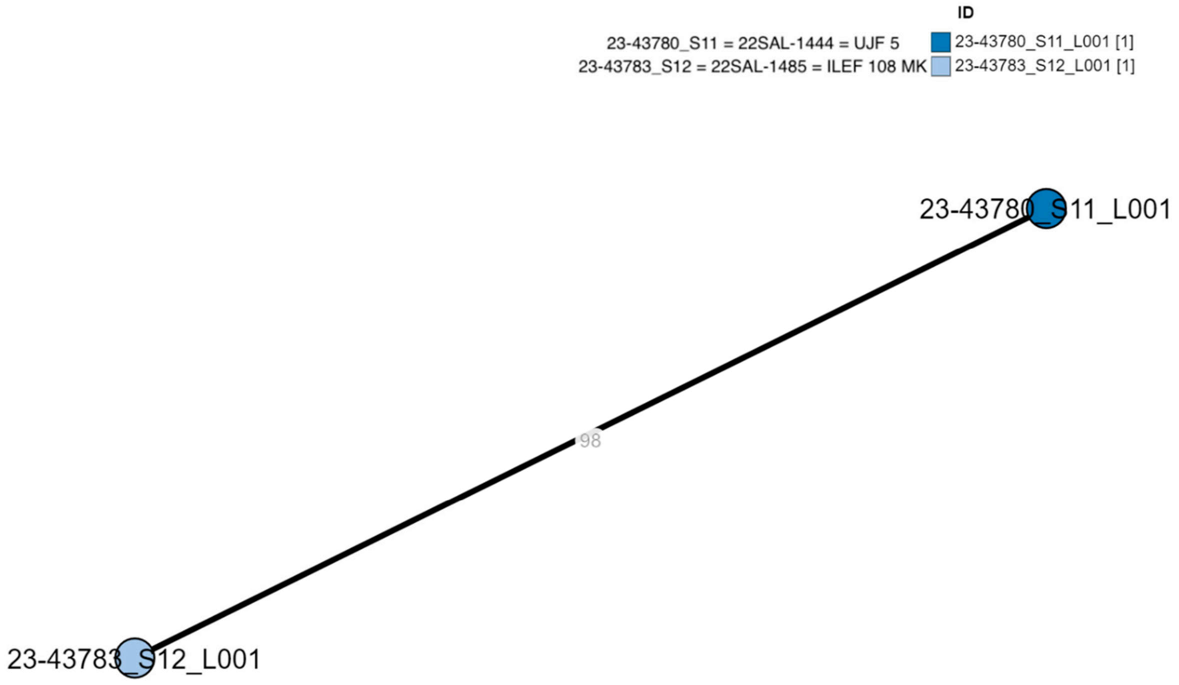

Figure S9: Cluster analysis for *Salmonella* Give isolated from poultry droppings in 2 markets in Kwara and Plateau States

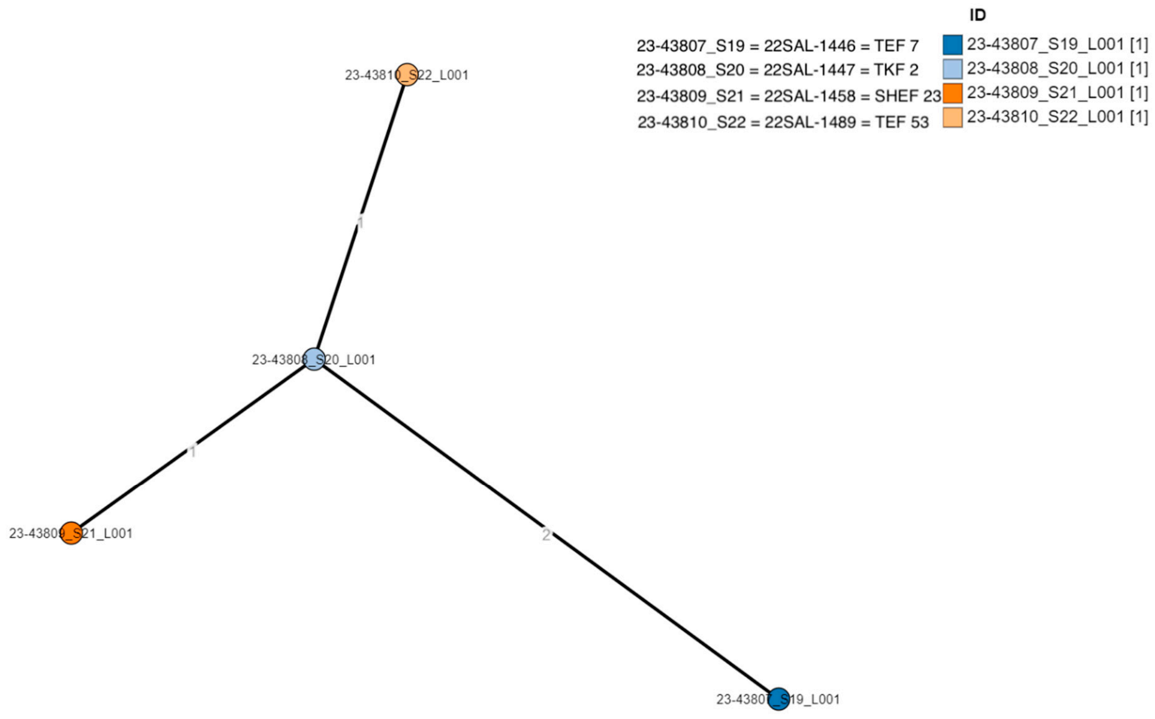

Figure S10: Cluster analysis for *Salmonella* Agama isolated from poultry droppings in 3 markets in Plateau State

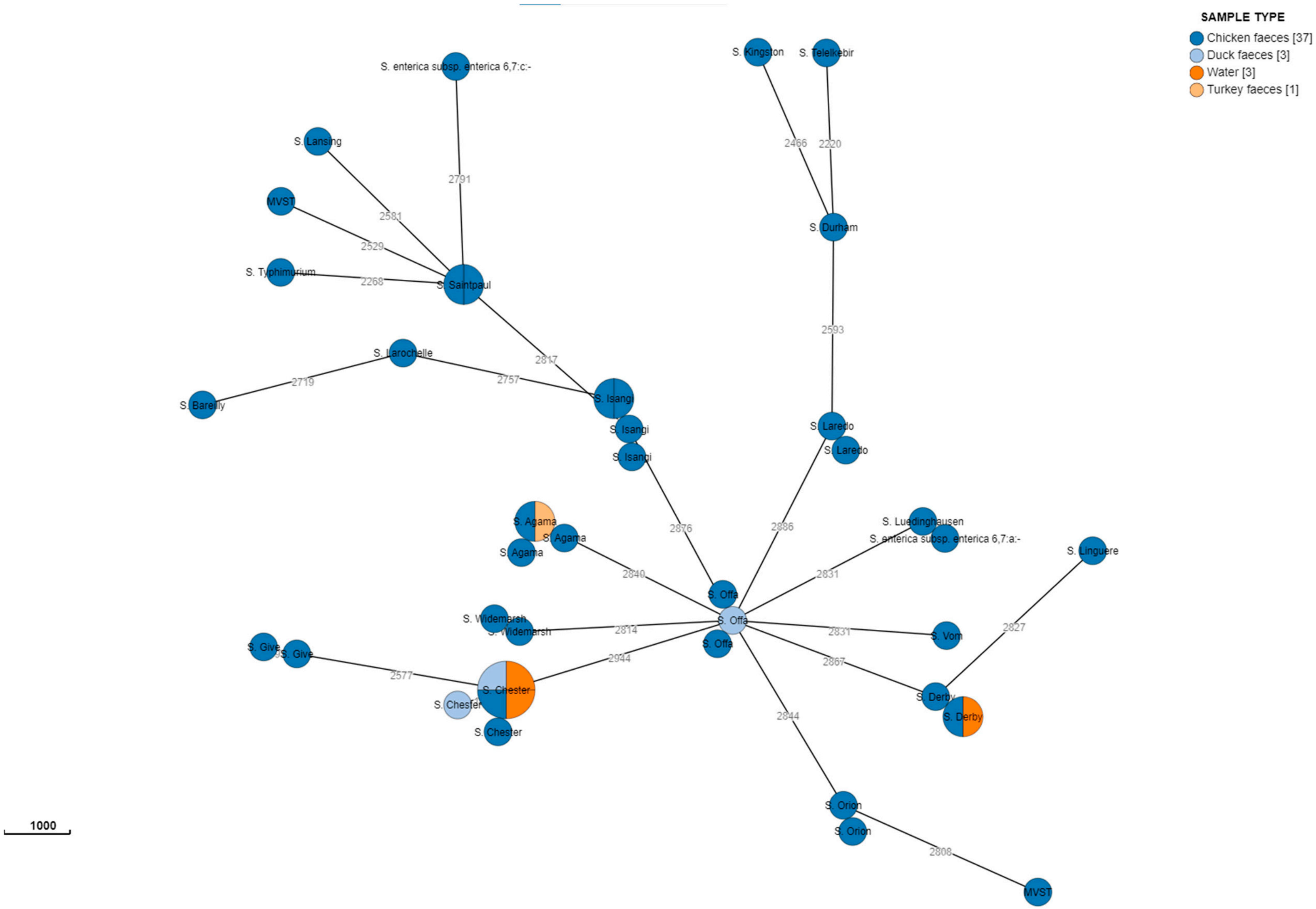
SAMPLE TYPE
Chicken faeces [37]
Duck faeces [3]
Water [3]
Turkey faeces [1]
S. enterica subsp. enterica 6,7:c:-
S. Lansing
MVST
S. Typhimurium
S. Saintpaul
S. Larochelle
S. Bareilly
S. Isangi
S. Isangi
S. Isangi
S. Kingston
S. Telelkebir
S. Durham
S. Laredo
S. Laredo
S. Agama
S. Agama
S. Agama
S. Luedinghausen
S. enterica subsp. enterica 6,7:a:-
S. Linguere
S. Offa
S. Offa
S. Offa
S. Widemarsh
S. Widemarsh
S. Vom
S. Give
S. Give
S. Chester
S. Chester
S. Chester
S. Derby
S. Derby
S. Orion
S. Orion
MVST
2791
2581
2529
2268
2817
2719
2757
2466
2220
2593
2876
2886
2840
2831
2814
2831
2827
2577
2944
2867
2844
2808
1000

Figure S11: Cluster analysis for the 44 strains based on sample type

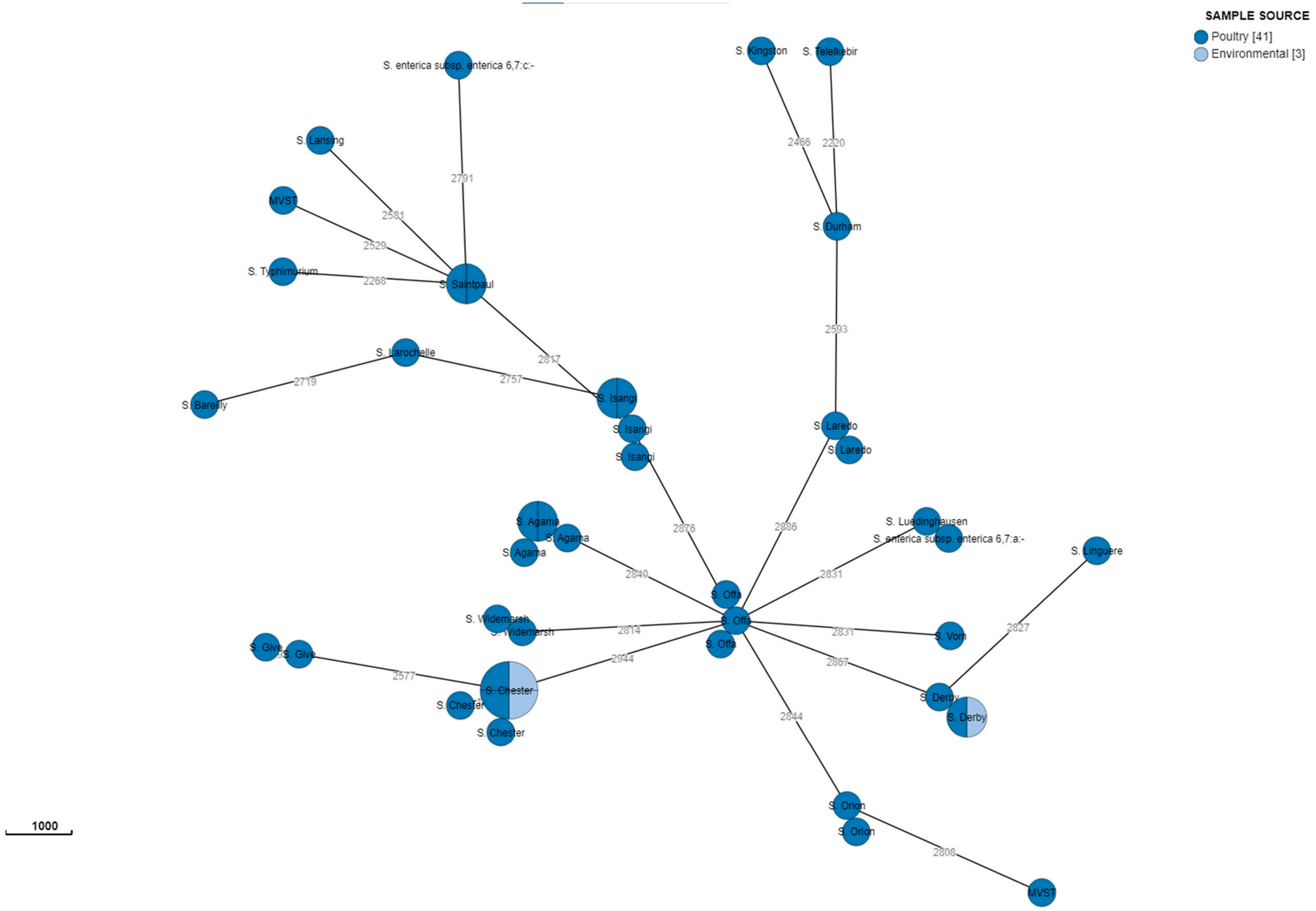

Figure S12: Cluster analysis for the 44 strains based on sample source
